# Endoscopic ultrasound with fine needle aspiration is useful in pancreatic cysts smaller than 3 cm

**DOI:** 10.1186/s12876-020-01565-9

**Published:** 2020-12-09

**Authors:** Sandra Faias, Marília Cravo, João Pereira da Silva, Paula Chaves, A. Dias Pereira

**Affiliations:** 1 Gastroenterology Department, Instituto Português de Oncologia de Lisboa Francisco Gentil EPE, Rua Prof Lima Basto, 1099-023 Lisbon, Portugal; 2 Faculty of Health Sciences, University of Beira Interior, Covilhã, Portugal; 3 Gastroenterology Department, Hospital Beatriz Ângelo, Av. Carlos Teixeira, 3, 2670-000 Loures, Portugal; 4 Faculty of Medicine, University of Lisbon, Lisbon, Portugal; 5 Pathology Department, Instituto Português de Oncologia de Lisboa Francisco Gentil EPE, Rua Prof Lima Basto, 1099-023 Lisbon, Portugal

**Keywords:** CEA, Pancreatic cyst, EUS-FNA, IPMN, MCN, Small size

## Abstract

**Background:**

In current guidelines, endoscopic ultrasound with fine-needle aspiration (EUS-FNA) is recommended in pancreatic cystic lesions (PCLs) with worrisome features (size ≥ 3 cm, mural nodule, or Wirsung dilation).

**Objective:**

To evaluate the diagnostic ability and assess the accuracy of EUS-FNA in PCLs smaller than 3 cm.

**Methods:**

Retrospective study of PCLs < 3 cm (2007–2016) undergoing EUS-FNA. Clinical, EUS and pancreatic cystic fluid (PCF) data were prospectively registered. Performance of EUS-FNA with PCF analysis for the detection of malignancy and accuracy in surgical cohort were analyzed.

**Results:**

We evaluated 115 patients with PCLs < 3 cm who underwent EUS-FNA. 19 patients underwent surgery, 7 had malignant, 8 pre-malignant, and the remaining 4 benign lesions. Mass/mural nodule was present in 27% of the cysts, CEA level was higher than 192 ng/mL in 39.4% of patients, and only 35% of cytologic samples were informative. Nevertheless, additional FNA for PCF analysis improved the diagnostic performance of EUS imaging—AUC = 0.80 versus AUC = 60.

**Conclusion:**

EUS-FNA has good accuracy in PCLs < 3 cm. It confirmed malignancy even in lesions without worrisome features (nodule/mass), with two in every five resections showing high-risk/malignant lesions. EUS-FNA was also useful to diagnose benign cysts, possibly allowing surveillance to be stopped in one in every five patients.

## Background

Pancreatic cancer is the 4th leading cause of cancer death in the USA and it is expected to be the 2nd by 2030. Mucinous pancreatic cysts are believed to be premalignant and would represent an excellent opportunity for early diagnosis in this malignancy [1].

The prevalence of pancreatic cysts over 1 cm in the general population is around 2% and cyst prevalence increases with age [2] making differential diagnosis of these lesions, a true challenge. Furthermore, the widespread use of abdominal imaging led to a significant increase in the diagnosis of asymptomatic pancreatic cystic lesions (PCLs) [3], including benign/inflammatory cysts. [e.g. serous cystadenomas (SCAs), pseudocysts], pre-malignant [intraductal papillary mucinous neoplasms (IPMNs) and mucinous cystic neoplasms (MCNs)], and malignant cysts [cystic adenocarcinomas (ADCs), cystic neuroendocrine tumors (NETs), acinar cell carcinomas, etc.].

The key question for pancreatic cyst management is to distinguish patients harboring advanced neoplasia who should be submitted to surgery, from those with pre-malignant lesions who require surveillance, and those with benign lesions who can be safely released from surveillance programs.

Imaging per se lacks accuracy for differential diagnosis of PCLs because there are no clear pathognomonic features of each cyst type, although some findings may suggest a particular diagnosis.

There is probably no other health disorder so prevalent and potentially severe, for which evidence is so low, due to the paucity of randomized trials performed. An attempt to summarize the best available evidence for the clinical management of PCLs has been made by experts in the field with the preparation of several recent guidelines [4–7].

In the absence of robust prospective data, current guidelines for management of PCLs are mostly driven by low-quality evidence, consensus, and opinion of experts [4–7]. Several of these guidelines [4, 5, 7] provide management guidance for PCLs with emphasis on high-risk features, including size greater than 3 cm, mural nodules, and dilation of the main pancreatic duct. The 2017 revised Fukuoka guidelines [5] propose using a cyst size greater than 3 cm as a worrisome feature to recommend EUS-FNA. The 2015 AGA guidelines [4] include cyst size greater than 3 cm as one of three high-risk features, along with dilation of the main pancreatic duct and presence of a mass or nodule, which should prompt an EUS-FNA, but only if two of these features are present simultaneously. Thus, in current clinical practice, for cysts greater than 3 cm, EUS-FNA is a reasonable next step.

Asymptomatic PCLs less than 3 cm without other worrisome features do not require EUS for evaluation. However, the described risk of malignancy in cysts smaller than 3 cm is about 6.5% and compares to 9% for cysts larger than 3 cm [8]. As such, the risk of missing malignancy or high-grade dysplasia in small pancreatic cysts should be considered. The superior imaging quality of EUS and additional FNA for pancreatic cyst fluid (PCF) analysis, including carcinoembryonic antigen (CEA) and cytology, may allow definitive cyst classification [9]. CEA allows distinction of mucinous cysts [10] and cytology, despite scant cellularity [11] and interobserver agreement limitations [12], may provide a definitive diagnosis of malignancy.

The aim of our study was to evaluate the diagnostic ability of EUS-FNA in pancreatic cysts smaller than 3 cm and assess its accuracy in small cysts referred for surgery.

## Methods

### Case selection

For this single-center retrospective study, we reviewed consecutive patients with PCLs submitted to EUS between 2007 and 2016, and selected all cysts smaller than 3 cm that were further evaluated with FNA, from our Endoscopic Ultrasound database and Pancreatic Cyst Registry, as approved by the Institutional Scientific Board and Ethics Committee (UIC/1143). For all patients, clinical data, EUS morphology, PCF analysis (CEA, amylase and cytology), clinical decision, and follow-up were prospectively collected and registered. All patients gave informed consent for EUS-FNA, standard PCF analysis, and residual PCF storage.

The main criterion for patient selection was having been submitted to EUS-FNA for evaluation of a pancreatic cyst smaller than 3 cm. Cysts were separated in two groups, A and B, respectively, according to the presence or absence of a solid component. Patients were divided into a surgical cohort, with definitive surgical pathology as reference standard for diagnosis, and a clinical cohort, with the diagnosis established by EUS-FNA with PCF analysis for CEA and/or cytology and morphologic stability after imaging surveillance for a minimum of six months.

EUS still-images were reviewed, with EUS findings, including cyst size, location, morphology (thick septa, wall thickening, or solid components, including mural nodule or mass that were defined as solid components inside or contiguous to the cyst, respectively), and main pancreatic duct features (dilation of 5–9 mm or cyst communication) retrieved from our database of prospectively collected data.

We did not perform contrast harmonic EUS (CH-EUS) and EUS-FNA was performed with a 22 or 25-gauge needle, as per attending physician decision, according to cyst location and morphology, namely size, presence of solid component to sample, and pancreatic duct dilation. Cysts were fully aspirated when possible, with prophylactic intravenous administration of ciprofloxacin during the procedure, followed by five days of oral administration, as suggested by the 2013 ASGE guidelines [13] effective during the study period, with antibiotic prophylaxis recommended before and maintained during 3 to 5 days after the procedure.

The PCF obtained was immediately centrifuged for cytospin preparation for cytological analysis, and the supernatant was sent for CEA (Architect, Abbott; chemiluminescent immunoassay) and amylase (Architect, Abbott; kinetic colorimetric method).

Cysts were classified as mucinous, indeterminate, or non-mucinous according to a CEA level ≥ 192 ng/mL, between 192 ng/mL and 5 ng/mL, or < 5 ng/mL, respectively. Cysts were further classified as benign serous cystadenomas (SCAs), in case of CEA level < 5 ng/mL with a matched or a non-diagnostic cytology. The cytological analysis of PCF, using the Papanicolaou Society of Cytopathology Guidelines [14], prompted cyst classification into three groups: (1) malignant cysts (MCs), having atypical or malignant cells and other neoplastic cells (cystic adenocarcinomas—ADCs, neuroendocrine tumors—NETs or solid pseudopapillary neoplasms-SPPNs); (2) pre-malignant cysts (PMCs) with mucinous benign epithelia without atypia or with low-grade atypia, including mucinous cystic neoplasms—MCNs and intraductal papillary neoplasms—IPMNs; and (3) benign cysts (BCs) with inflammatory cells, neoplastic benign non-mucinous cells, or other neoplastic cells, for example SCAs, pseudocysts, lymphangiomas.

After undergoing EUS-FNA, patients were referred for surgery (surgical cohort) or surveillance, palliation, or endoscopic treatment (clinical cohort), according to the consensus guidelines of Sendai 2006 [15] revised in Fukuoka in 2012 [16] and attending physician’s decision. Magnetic resonance imaging (MRI) or EUS were used in surveillance of the clinical cohort. The diagnostic accuracy of EUS-FNA was evaluated in the surgical cohort.

### Statistical analysis

Descriptive data were expressed as mean ± SD or median, and range. Chi-square test or Fisher’s exact test were used to assess differences between cysts requiring surgery or surveillance, for dichotomous variables, and student *t* test or the Wilcoxon rank-sum test for continuous variables. A statistical significance was defined as a *p* value < 0.05. The sensitivity, specificity, positive and negative predictive values, and diagnostic accuracy of EUS imaging, CEA level, and cytology in PCF were evaluated for the diagnosis of high-risk/malignant cysts in the surgical cohort. Receiver operator curves were generated, and area under the curve (AUC) was calculated. Statistical analysis was performed using SPSS Statistics version 23 (IBM Corp, Armonk, NY).

## Results

### Demographics and cyst characteristics

Of 167 patients referred to EUS for evaluation of PCLs smaller than 3 cm, FNA was not performed in 52 due to difficult location, small size, no worrisome features, or no visualization of the lesion. We present the features of FNA and non-FNA cohorts in Additional file 1: Table S1.

We further evaluated 115 patients with small cysts who underwent FNA for PCF analysis, including the surgical and clinical cohorts with 19 and 96 patients, respectively. Table 1 shows clinical, endosonographic, and PCF analysis features of all patients included. There were 49/115 (42.6%) PCLs that were 2 cm or more in size and there were no patients with Wirsung dilation. Reasons for undergoing EUS-FNA included an initial diagnostic evaluation in 93 (80.8%), a change in cyst morphology during surveillance in 11 (9.6%), and was not described in 11 (9.6%) patients. No adverse events related to the procedure were recorded in the present series.

**Table 1:** Demographics, cyst morphological features, and PCF analysis

*Patients, n*	*115*
Females, n (%)	75 (65%)
Mean age, years	
Mean ± standard deviation (range)	63 ± 12 (33–86)
F-up time, months	
Mean ± standard deviation (range)	37 ± 30 (6–134)
Symptoms, n (%)	21 (18.3%)
*Cysts, n*	*115*
Location n, (%)	
Head	45 (39.1%)
Body	42 (36.6%)
Tail	23 (20%)
Multiple	5 (4.3%)
Size, mm	
Mean ± standard deviation (range)	19 ± 6 (5–29)
Size, n (%)	
≤ 10 mm	10 (8.7%)
10–20 mm	56 (48.7%)
≥ 20 mm	49 (42.6%)
Mural nodule/mass, n (%)	31 (27%)
Mural nodule/mass, size (mm)	
Mean ± standard deviation (range)	16.2 ± 12.2 (2–49)
Size > 10 mm, n (%)^a^	13 (52%)
Wirsung dilation, n (%)	0 (0%)
*PCF analysis*	
*CEA*, n (%)^b^	
≤ 5 ng/mL	18 (18.2%)
5–192 ng/mL	42 (42.4%)
≥ 192 ng/mL	39 (39.4%)
*Amylase*, n (%)^c^	
< 250 UI/mL	38 (39.6%)
≥ 250 UI/mL	50 (60.4%)
*Cytology*	
Acellular	75 (65.2%)
Benign or inflammatory	20 (17.4%)
LGD	3 (2.6%)
Malignant, atypical, NET	17 (14.8%)
*Clinical decision after EUS-FNA*, n (%)	
Surgery	19 (16.5%)
Imaging surveillance	80 (69.6%)
Palliation^d^	5 (4.3%)
Lost to follow-up	11(9.6%)

^a^Nodule/mass size available in 25 patients

^b^CEA available in 99 patients

^c^Amylase available in 96 patients

^d^Bad surgical candidates (2)/unresectable concomitant ADCs (3)

By combining CEA level and cytology results in PCF, we found that in 18 PCLs with CEA level ≤ 5 ng/mL, cytology identified 1 malignant, 1 NET, and 3 benign lesions. For the 42 PCLs with a CEA level between 5 and 192 ng/mL, cytology identified 2 atypical, 2 NETs, and 6 benign lesions. For the 39 PCLs with a CEA ≥ 192 ng/mL, cytology identified 4 malignant, 7 mucinous (including 3 samples with low-grade atypia), and 6 benign lesions. Considering cytology as the pre-surgical gold standard of malignancy, CEA values had considerable overlap in malignant and non-malignant cysts, without discriminative power (*p* = 0.053).

### Surgical pathology diagnosis and EUS-FNA diagnostic performance

**Fig. 1:**
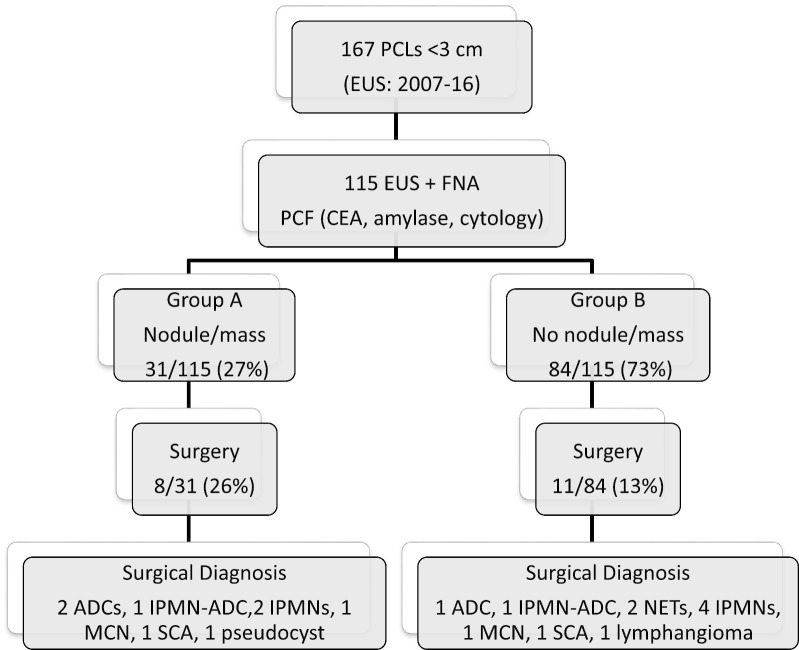
Flowchart with the pancreatic cysts studied by EUS-FNA and the diagnosis of 19 pancreatic cystic lesions that underwent surgery. *ADCs* adenocarcinomas, *IPMNs* intraductal papillary mucinous neoplasms, *NETs* neuroendocrine tumors, *MCNs* mucinous cystic neoplasms, *SCAs* serous cystadenomas

Figure 1 shows the discriminative power of nodule/mass within the cyst in surgical patients. The overall rate of surgery in patients with cysts smaller than 3 cm evaluated with FNA was 17% (19/115). In the subgroup of 31 patients with a concomitant mass or nodule (Group A), the rate of surgery was 26% (8/31), while in the subgroup of 84 patients without mural nodule or mass (Group B), the surgery rate was 13% (11/84), *p* value of 0.092. High-risk/malignant lesions were present in Group A (n = 3) and Group B (n = 4). Detailed data of lesions that underwent surgery are shown in the Additional file 1: Table S2. Broadly, 7 malignant, 8 pre-malignant, and 4 benign PCLs were resected. The 7/19 (37%) malignant or high-risk lesions resected included cystic ADCs (3), NETs (2), and IPMNs-ADC (2). As shown in Table 2, the accuracy of EUS imaging was improved by PCF analysis for cytological diagnosis of malignant cysts.

**Table 2:** Performance characteristics of EUS imaging, PCF fluid analysis (CEA and cytology), and EUS-FNA results (imaging, CEA and cytology) for malignant cysts identification

Malignant cysts (7/19)	Sensitivity (95% CI)	Specificity (95% CI)	PPV (95% CI)	NPV (95% CI)	Accuracy (95% CI)	Area under the ROC (CI)
EUS-imaging	38 (9–76)	55 (23–83)	33 (14–59)	60 (41–76)	48 (25–72)	0.60 (0.30–0.90)
EUS-FNA (CEA + cytology)	86 (42–99)	50 (21–79)	50 (35–66)	86 (47–98)	63 (38–84)	0.80 (0.58–1.00)

*EUS* endoscopic ultrasound, *FNA* fine-needle aspiration, *PPV* positive predictive value, *NPV* negative predictive value, *CI* confidence interval, *ROC* receiver-operating characteristics

Table 3 compares demographic, clinical, and cystic features of patients harboring malignant/high risk and non-malignant lesions. Symptomatic lesions, mostly presenting with non-specific abdominal pain (Additional file 1: Table S2), with a larger size and presenting a nodule/mas or suspicious lymph nodes, with a conclusive cytology, were more often malignant. Mural nodules were relevant for diagnosis of mucinous malignant lesions, but not for other rare types of high-risk lesions (e.g. cystic NETs and ADCs), in which a thick wall justified FNA, with cytology rendering the final diagnosis. Although mural nodules correlate with malignancy, they are not pathognomonic, as they also occur in low-risk lesions. Small cysts without worrisome features in patients with non-specific abdominal may correspond to malignant/high-risk lesions requiring surgery (Patients 3, 8, and 9 in Additional file 1: Table S2).

**Table 3:** Demographics and cystic features in malignant versus non-malignant cysts in both cohorts

	Malignant (n = 17)	Non-malignant (n = 98)	*p* value
Female n (%)	9 (52.6%)	65 (66.3%)	0.551
Mean age ± SD (range)	62.9 ± 12.3 (43–80)	63.1 ± 11.9 (33–86)	0.987
Symptoms^a^ n (%)	12 (70.6%)	9 (9.2%)	0.000
Cyst location (head, body, tail, multiple)	10/5/2/0	35/37/21/5	0.059
Cyst size (mm) mean ± SD (range)	21.6 ± 6 (10–29)	18 ± 6.1 (5–29)	0.030
Cyst size > 20 mm	10 (58.8%)	33 (33.7%)	0.049
Septa n (%)	6 (35.3%)	56 (57.1%)	0.080
Nodule n (%)	10 (58.8%)	21 (21.4%)	0.001
Adenopathy n (%)	6 (35.3%)	1 (1%)	0.000
Amylase (U/L) ± SD (range)	3564 ± 10,644 (7–40,223)	41,437 ± 111,030 (3–786,486)	0.049
CEA (ng/mL) ± SD (range)	1522 ± 39,505 (5–150,490)	2725 ± 17,173 (1–155,012)	0.053
Conclusive cytology n (%)	14 (82.4%)	25 (25.8%)	0.001

*SD* standard deviation

^a^Pain, weight loss, vomiting, jaundice, acute pancreatitis

## Discussion

Our study endorses EUS-FNA with PCF analysis in cysts smaller than 3 cm, even if additional worrisome features (mural nodule/mass) are absent, due to its ability to diagnose malignant small PCLs pre-operatively. In our surgical series we found malignant and pre-malignant lesions in 15/115 patients (13%), which is similar to the rate of malignancy in lesions greater than 3 cm [17]. Combined EUS-FNA has better performance than isolated EUS imaging for malignant cyst diagnosis, with an area under the curve of 0.8 (95% CI 0.58–1).

In our series, we had 21/115 (18.3%) of symptomatic patients, with a striking difference in malignancy rates between symptomatic 12/17 (70.6%) and 9/98 (9.2%) asymptomatic patients, *p* < 0.0001. This finding highlights that EUS and FNA features should always be used in conjunction with clinical findings, as well as laboratory and other imaging techniques, in order to improve the differential of PCLs. In our cohort of 19 surgical resected cysts, we found 7/19 (37%) histologically high-risk/malignant cysts, including NETs, IPMN-associated ADCs, and cystic ADCs. This rate of malignancy in small cysts is similar to 35.5% of histologically malignant cysts in lesions larger than 3 cm, as reported by Chebib et al. [17] and higher than other surgical series, with 16% of high-risk malignant IPMNs, as reported by Ridtitid et al. [18], 32% by Singhi et al. [19], and 29% by Lekkerkeker et al. [20]. Furthermore, 8/19 (42%) patients had pre-malignant cysts. Our results support the concept that size by itself should not be a decisive factor to perform or not FNA.

In order to select high-risk/malignant small cysts in this series, the presence of a nodule per se was not particularly helpful, with a 10% malignancy rate in resected cysts in Group A (with mural nodule/mass) versus 5% in Group B (without nodule/mass), *p* = NS. This would apparently support the recommendation of AGA guidelines requiring at least two worrisome features for further evaluation of PCLs [4]. However, we had 5% of small cysts without worrisome features that were confirmed to be high-risk or malignant in surgical pathology specimens, including two NETs and one cystic ADC, that presented with non-specific abdominal pain.

In a recent meta-analysis evaluating risk factors for malignancy and high-grade dysplasia exclusively in IPMNs, the presence of a nodule was considered relevant, but not cyst size [21]. Our study includes relevant PCLs besides IPMNs, particularly two cystic NETs and a cystic ADC without associated nodules, that possibly explain the discrepancies in mural nodule significance with the previous study and current guidelines, that refer only to BD-IPMNs [5] and cannot be applied in other cyst types (e.g. cystic NET). Our study represents current clinical practice, includes an heterogeneous group of cysts (NETs, IPMNs, SCAs, MCNs, etc.), in which current guidelines can be misleading, as clinical decisions rely on a presumed diagnosis, that even with additional FNA for PCF analysis, seldom allows a definitive diagnosis. Our results are in line with previous publications [19, 22] and expert opinions [23], that AGA guidelines [4] may be imprecise in discriminating between neoplastic and non-neoplastic cysts, and of limited value in early detection of pancreatic cancer. More recent guidelines, recommending EUS-FNA for PCF analysis in indeterminate cysts, are probably more adequate for this purpose [6].

Additionally, in our series there were 18% (18/99) of patients with non-mucinous cysts after EUS-FNA (CEA level ≤ 5 ng/mL), supporting a strategy to stop surveillance. However, in these 18 patients cytology identified 1 malignant cyst and 1 NET, further reinforcing the value of cytology to definitely exclude malignancy. Similarly, there were 42% (42/99) of PCLs with a CEA level between 5 and 192 ng/mL, considered indeterminate for mucinous cyst diagnosis, with cytology diagnosing 4 high risk/malignant lesions—2 atypical and 2 NETs. Using CEA level ≥ 192 ng/mL as cut-off for mucinous cysts diagnosis would reduce sensitivity and exclude several mucinous lesions from surveillance. This imperfect performance of CEA highlights the need of better biomarkers in PCLs. PCF glucose may be more advantageous than CEA in routine diagnosis of small pancreatic mucinous cysts, reducing “indeterminate” diagnosis and requiring minimal amount of PCF as shown by others and ourselves in a recently published study [24–26].

The value of EUS in diagnosis and staging of malignancy and its clinical impact on patient management has been previously established [27]. Moreover, CH-EUS is a useful adjunct in the differential of pancreatic cysts [28] that shows malignant vegetations as solid components with hyperenhancement, thus allowing to direct EUS-FNA to potential neoplastic areas while avoiding puncture of debris and mucus plugs. CH-EUS allows the differentiation between pseudocysts and other PCLs, but not between MCNs and SCAs. Therefore CH-EUS does not replace FNA and should be used in conjunction with clinical, laboratory, and other imaging techniques in the differential diagnosis of PCLs.

No adverse events occurred in our study. We did not transverse more that 10 mm of pancreatic parenchyma or crossed the wirsung duct to avoid pancreatitis. Besides, cysts were fully aspirated when possible and prophylactic intravenous administration of ciprofloxacin during the procedure, was followed by 5 days of oral administration in all cases, as per 2013 ASGE guidelines [13]. These were effective during the study period, and recommended antibiotic prophylaxis before and 3 to 5 days after EUS-FNA, although the conflicting evidence. Recent evidence from a randomized trial published in 2020, showed that antibiotic prophylaxis might not be needed at all before EUS-FNA of pancreatic cysts [29].

Our study has several strengths. The main is to evaluate PCLs assessed by EUS-FNA as standard of care including predominantly low-risk PCLs, better representing daily practice. Moreover, the patient and cyst data were prospectively collected and registered with most PCLs with CEA and cytology evaluation. We had CEA level for most PCLs, but cytology was informative in only 33% of patients, which compares to 76% in lesions larger than 3 cm [17].

The limitations of our study include its retrospective design, which may have introduced unintended biases, the modest sample size, and the low number of surgical pathology diagnoses. Also, we evaluated cyst morphology by EUS, not by other imaging methods because several patients were outside referrals without images available for review. Finally, we studied diagnostic accuracy of EUS-FNA exclusively in resected cysts due to possible diagnostic uncertainty of the clinical cohort, in which diagnosis relied on clinico-cytological features.

In summary, when debating the role of EUS-FNA in pancreatic cysts smaller than 3 cm, one can support either side of the coin. On the one hand, we found most PCF analysis inconclusive (more than 40% of CEA levels between 5 and 192 ng/mL, and overall two thirds of acellular samples) making EUS-FNA an invasive and often unhelpful technique. On the other, EUS-FNA allowed to diagnose malignancy in some patients who would otherwise be surveilled, potentially improving outcome and cost-effectiveness of the program. Mass/nodules were helpful for malignant cyst diagnosis, although absent in cystic NETs and cystic PDACs with central necrosis, while often occurring in non-high-risk IPMNs. As the performance of any isolated marker is imperfect, according to our results, combining clinical, morphologic, biochemical, and cytological data significantly improves the diagnosis of malignancy. Furthermore, EUS-FNA diagnosed benign cysts in almost 1 in every 5 patients, allowing their release from invasive and costly surveillance programs. Surveillance is especially important in young and healthy patients, while discontinuation is advisable in elderly individuals with increased risk of death from other causes than pancreatic cancer.

## Conclusion

EUS-FNA allows pre-operative diagnosis of small PCLs harboring malignancy. Finding new and more specific biomarkers may also enhance this strategy.

## Supplementary Information

**Additional file 1.** **Table S1.** Demographics and cystic features in FNA vs. non-FNA cohorts in cysts smaller than 3 cm. **Table S2.** Clinical, imaging, biochemical, cytologic features and final diagnosis of 19 resected cysts.

## Data Availability

All available in an additional file.

## References

[1] Lennon AM, Wolfgang CL, Canto MI (2014). The early detection of pancreatic cancer: What will it take to diagnose and treat curable pancreatic neoplasia?. Cancer Res.

[2] Kromrey M, Bülow R, Hübner J (2017). Prospective study on the incidence, prevalence and 5-year pancreatic-related mortality of pancreatic cysts in a population-based study. Gut.

[3] Stark A, Donahue TR, Reber HA, Hines OJ (2016). Pancreatic cyst disease: a review. JAMA.

[4] Vege SS, Ziring B, Jain R, Moayyedi P (2015). American Gastroenterological Association Institute guideline on the diagnosis and management of asymptomatic neoplastic pancreatic cysts. Gastroenterology.

[5] Tanaka M, Castillo F-D, Kamisawa T (2017). Revisions of international consensus Fukuoka guidelines for the management of IPMN of the pancreas. Pancreatology.

[6] Elta GH, Enestvedt BK, Sauer BG, Lennon AM (2018). ACG clinical guideline: diagnosis and management of pancreatic cysts. Am J Gastroenterol.

[7] Del Chiaro M, Besselink MG, Scholten L (2018). European evidence-based guidelines on pancreatic cystic neoplasms. Gut.

[8] Sahora K, Mino-Kenudson M, Brugge W (2013). Branch duct intraductal papillary mucinous neoplasms: does cyst size change the tip of the scale? A critical analysis of the revised international consensus guidelines in a large single-institutional series. Ann Surg.

[9] Wang QX, Xiao J, Orange M (2015). EUS-guided FNA for diagnosis of pancreatic cystic lesions : a meta-Analysis. Cell Physiol Biochem.

[10] Brugge WR, Lewandrowski K, Lee-Lewandrowski E (2004). Diagnosis of pancreatic cystic neoplasms: a report of the cooperative pancreatic cyst study. Gastroenterology.

[11] Morales-Oyarvide V, Yoon WJ, Ingkakul T (2014). Cystic pancreatic neuroendocrine tumors: the value of cytology in preoperative diagnosis. Cancer (Cancer Cytopathol).

[12] Pitman MB, Centeno BA, Genevay M (2013). Grading epithelial atypia in endoscopic ultrasound-guided fine-needle aspiration of intraductal papillary mucinous neoplasms: an international interobserver concordance study. Cancer Cytopathol.

[13] Khashab MA, Chithadi KV, Acosta RD (2015). Antibiotic prophylaxis for GI endoscopy. Gastrointest Endosc.

[14] Pitman MB, Centeno BA, Ali SZ (2014). Standardized terminology and nomenclature for pancreatobiliary cytology: the Papanicolaou Society of Cytopathology guidelines. Cytojournal.

[15] Tanaka M, Chari S, Adsay V (2006). International consensus guidelines for management of intraductal papillary mucinous neoplasms and mucinous cystic neoplasms of the pancreas. Pancreatology.

[16] Tanaka M, Castillo CF, Adsay V (2012). International consensus guidelines for the management of IPMN and MCN of the pancreas. Pancreatology.

[17] Chebib I, Yaeger K, Mino-kenudson M, Pitman MB (2014). The role of cytopathology and cyst fluid analysis in the preoperative diagnosis and management of pancreatic cysts > 3 cm. Cancer (Cancer Cytopathol).

[18] Ridtitid W, Dewitt JM, Schmidt CM (2016). Management of branch-duct intraductal papillary mucinous neoplasms: a large single-center study to assess predictors of malignancy and long-term outcomes. Gastrointest Endosc..

[19] Singhi AD, Zeh HJ, Brand RE (2018). American Gastroenterological Association guidelines are inaccurate in detecting pancreatic cysts with advanced neoplasia : a clinicopathologic study of 225 patients with supporting molecular data. Gastrointest Endosc.

[20] Lekkerkerker SJ, Besselink MG, Busch OR (2017). Comparing 3 guidelines on the management of surgically removed pancreatic cysts with regard to pathological outcome. Gastrointest Endosc.

[21] Marchegiani G, Andrianello S, Borin A (2018). Systematic review, meta-analysis, and a high volume center experience supporting the new role of mural nodules proposed by the updated 2017 international guidelines on IPMN of the pancreas. Surgery..

[22] Sahar N, Razzak A, Kanji ZS (2018). New guidelines for use of endoscopic ultrasound for evaluation and risk stratification of pancreatic cystic lesions may be too conservative. Surg Endosc.

[23] Canto MI, Hruban RH (2015). Managing pancreatic cysts: less is more?. Gastroenterology.

[24] Zikos T, Pham K, Bowen R (2015). Cyst fluid glucose is rapidly feasible and accurate in diagnosing mucinous pancreatic cysts. Am J Gastroenterol.

[25] Carr RA, Yip-Schneider MT, Simpson RE (2017). Pancreatic cyst fluid glucose: rapid, inexpensive, and accurate diagnosis of mucinous pancreatic cysts. Surgery.

[26] Faias S, Pereira L, Roque R (2019). Excellent accuracy of glucose level in cystic fluid for diagnosis of pancreatic mucinous cysts. Dig Dis Sci.

[27] Fusaroli P, Kypraios D, Eloubeidi MA, Caletti G (2012). Levels of evidence in endoscopic ultrasonography: a systematic review. Dig Dis Sci.

[28] Fusaroli P, Serrani M, De Giorgio R (2016). Contrast harmonic-endoscopic ultrasound is useful to identify neoplastic features of pancreatic cysts (with videos). Pancreas.

[29] Colán-Hernández J, Sendino O, Loras C (2020). Antibiotic prophylaxis is not required for endoscopic ultrasonography-guided fine-needle aspiration of pancreatic cystic lesions, based on a randomized trial. Gastroenterology.

