# Supplementary material for: Endoscopic ultrasound with fine needle aspiration is useful in pancreatic cysts smaller than 3 cm
Source: BMC Gastroenterol. 2020 Dec 9;20:413. doi: 10.1186/s12876-020-01565-9 (PMC7727209; doi:10.1186/s12876-020-01565-9)
Supplement: Supplementary file 1 — Additional file 1. Table S1. Demographics and cystic features in FNA vs. non-FNA cohorts in cysts smaller than 3 cm. Table S2. Clinical, imaging, biochemical, cytologic features and final diagnosis of 19 resected cysts. [file 12876_2020_1565_MOESM1_ESM.docx]

| Table S1. Demographics and cystic features in FNA vs. non-FNA cohorts in cysts smaller than 3 cm. | | | |
| --- | --- | --- | --- |
|  | **FNA (n=115)** | **Non-FNA (n=52)** | ***p* value** |
| Female n (%) | 75 (%) | 32 (%) | 0.646 |
| Mean age ± SD (range) | 63.1±11.9 (33-86) | 64.9±13.5 (30-83) | 0.08 |
| Symptoms* n (%) | 21 (18.3%) | 3 (5.8%) | 0.092 |
| Cyst location (head, body, tail, multiple) | 45/42/23/5 | 24/17/5/6 | 0.128 |
| Cyst size (mm) mean ± SD (range) | **18.5±6.2 (5-21)** | **13.1±5.9(4-27)** | **<0.0001** |
| Cyst size >20 mm | **43 (37.4%)** | **6 (11.5%)** | **<0.0001** |
| Septa n (%) | **62/113(53.9%)** | **12 (23.1%)** | **<0.0001** |
| Nodule n (%) | **31 (26.9%)** | **3 (5.8%)** | **0.001** |
| Adenopathy n (%) | 8 (6.9%) | 2 (3.8%) | 0.658 |
| SD, standard deviation; * pain, weight loss, vomiting, jaundice, acute pancreatitis | | | |

| Table S2. Clinical, imaging, biochemical, cytologic features and final diagnosis of 19 resected cysts. | | | | | | | | | |
| --- | --- | --- | --- | --- | --- | --- | --- | --- | --- |
| Pt | Gender, age(y) | Symptoms | EUS:  Cyst, nodule(cm) | Imaging:  Diagnosis | FNA  CEA (ng/mL) | FNA  cytology** | FNA Diagnosis  (CEA+cytology) | Surgical pathology | Final Diagnosis |
| 1 | F, 74 | New-onset diabetes | 2, Un | High-risk | 2297 | VI.Malignant | Malignant | ADC | Malignant |
| 2 | F, 55 | Pain | 2.5, 4 | High-risk | 15306 | IV.Neoplastic: Other | Pre-malignant | IPMN-ADC | Malignant |
| 3 | F, 47 | Pain | 1.5, No | Low-risk | 24 | IV.Neoplastic: Other | Malignant | NET | Malignant |
| 4 | F, 68 | No | 2, No | Low-risk | Thick | IV.Neoplastic: Other | Pre-malignant | IPMN | Pre-malignant |
| 5 | M, 72 | Jaundice | 1.5, No | Low-risk | 88 | I.Non-diagnostic | Un | ADC | Malignant |
| 6 | F, 35 | No | 1.7, No | Low-risk | 45 | IV.Neoplastic: Benign | Benign | Lymphangioma | Benign |
| 7 | M, 77 | Pain | 1.5, No | Low-risk | 207 | IV.Neoplastic: Other | Pre-malignant | IPMN | Pre-malignant |
| 8 | F, 57 | Pain | 2.3, No | Low-risk | 5 | IV.Neoplastic: Other | Malignant | NET | Malignant |
| 9 | M, 75 | Pain | 2, No | Low-risk | 10003 | V.Suspicious | Malignant | IPMN-ADC | Malignant |
| 10 | M, 49 | No | 2.5, 1.5 | High-risk | 19 | I.Non-diagnostic | Un | Pseudocyst | Benign |
| 11 | F, 56 | Pain | 1.7, No | Low-risk | 1759 | IV.Neoplastic: Other | Pre-malignant | MCN | Pre-malignant |
| 12 | F, 37 | No | 2.4, 0.6 | High-risk | 305 | IV.Neoplastic: Other | Pre-malignant | MCN | Pre-malignant |
| 13 | M, 67 | Weight loss | 1.5, Un | High-risk | 574 | IV.Neoplastic: Other | Pre-malignant | IPMN | Pre-malignant |
| 14 | M, 55 | No | 2.7, 0.9 | High-risk | 18 | I.Non-diagnostic | Un | SCA | Benign |
| 15 | F, 51 | Pain | 2, 2 | High-risk | Un* | V.Suspicious | Malignant | IPMN | Pre-malignant |
| 16 | F, 64 | **No** | 2.9, No | Low-risk | 1 | VI.Malignant | Malignant | SCA | Benign |
| 17 | F, 63 | No | 2.4, No | Low-risk | 356 | V.Suspicious | Malignant | IPMN | Pre-malignant |
| 18 | F, 77 | No | 1.8, No | Low-risk | 999 | IV.Neoplastic: Other | Pre-malignant | IPMN | Pre-malignant |
| 19 | F, 73 | Pain | 2.8, 1.2 | High-risk | 2600 | V.Suspicious | Malignant | ADC | Malignant |
| Pt, patient; F, female; M, male; Un-unknown size;*Thick fluid; **According to the Papanicolaou Society Guidelines | | | | | | | | | |
